# Cannabinoids, Blood–Brain Barrier, and Brain Disposition

**DOI:** 10.3390/pharmaceutics12030265

**Published:** 2020-03-15

**Authors:** Fabrizio Calapai, Luigi Cardia, Emanuela Elisa Sorbara, Michele Navarra, Sebastiano Gangemi, Gioacchino Calapai, Carmen Mannucci

**Affiliations:** 1 Department of Biomedical and Dental Sciences and Morphological and Functional Imaging, University of Messina, 98125 Messina, Italy; f.calapai@gmail.com (F.C.); emanuela.sorbara@hotmail.it (E.E.S.); cmannucci@unime.it (C.M.); 2 Anesthesia, Intensive Care and Pain Therapy, A.O.U.G. Martino Messina, University of Messina, 98125 Messina, Italy; luigicardia1@gmail.com; 3 Department of Chemical, Biological, Pharmaceutical and Environmental Sciences, University of Messina, 98166 Messina, Italy; mnavarra@unime.it; 4 School and Division of Allergy and Clinical Immunology, Department of Experimental Medicine, University of Messina, 98125 Messina, Italy; gangemis@unime.it

**Keywords:** cannabinoids, blood–brain barrier, THC, CBD, delta-9-tetrahydrocannabinol, cannabidiol, brain delivery, brain disposition

## Abstract

Potential therapeutic actions of the cannabinoids delta-9-tetrahydrocannabinol (THC) and cannabidiol (CBD) are based on their activity as analgesics, anti-emetics, anti-inflammatory agents, anti-seizure compounds. THC and CBD lipophilicity and their neurological actions makes them candidates as new medicinal approaches to treat central nervous system (CNS) diseases. However, they show differences about penetrability and disposition in the brain. The present article is an overview about THC and CBD crossing the blood–brain barrier (BBB) and their brain disposition. Several findings indicate that CBD can modify the deleterious effects on BBB caused by inflammatory cytokines and may play a pivotal role in ameliorating BBB dysfunction consequent to ischemia. Thus supporting the therapeutic potential of CBD for the treatment of ischemic and inflammatory diseases of CNS. Cannabinoids positive effects on cognitive function could be also considered through the aspect of protection of BBB cerebrovascular structure and function, indicating that they may purchase substantial benefits through the protection of BBB integrity. Delivery of these cannabinoids in the brain following different routes of administration (subcutaneous, oral, and pulmonary) is illustrated and commented. Finally, the potential role of cannabinoids in drug-resistance in the clinical management of neurological or psychiatric diseases such as epilepsy and schizophrenia is discussed on the light of their crossing the BBB.

## 1. Introduction

**Figure 1:**
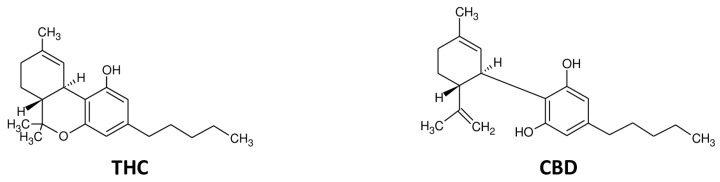
Chemical structure of delta-9-tetraidrocannabinolo (THC) e cannabidiol (CBD).

Research on cannabinoids shows that these compounds have a number of pharmacobiological effects, such as hypothermia, catalepsy, anti-inflammatory activity, analgesia, induction of cell proliferation, growth arrest, or apoptosis [1,2]. More than one hundred cannabinoids have been identified in the plant cannabis. However, the most known are delta-9-tetrahydrocannabinol (THC) and cannabidiol (CBD) having the same molecular formula and weight (C_21_H_30_O_2_; molecular weight 314.5 g/mol) and poor solubility in water, but good solubility in most organic solvents, such as alcohol and lipids [3] (Figure 1). Apart from THC and CBD, partially abundant phytocannabinoids are cannabinol and cannabichromene (CBC), cannabidivarine, delta-9-tetrahydrocannabivarine, and cannabigerol. THC and CBD share the same plant derivation but show distinct biological effects [4,5]. THC is considered the main psychoactive cannabinoid, while CBD is not psychoactive and attenuates THC behavioral and metabolic effects [6]; on the contrary, CBD produces anxiolytic and antipsychotic effects and is thought to attenuate the psychotropic activity of THC [7].

The phytocannabinoid THC binds to cannabinoid receptors. Cannabinoid receptors 1 (CB1R) are a G protein-coupled receptors (GPCR) type, widely located in neurons and glial cells [8]. CB1R is involved in the modulation of neurotransmissions, and its activation is responsible for the psychotropic cannabinoids’ effects. Cannabinoid receptors 2 (CB2R) are GPCR expressed by hematopoietic cells and moderately expressed in specific brain areas and peripheral cells. CB2R is located principally in cells involved in immune activity, and when activated it can participate in anti-inflammatory and immunomodulatory response. Stimulation of CB2R is followed by activation of molecular communication pathways equivalent to those triggered for CB1R [9,10].

THC is a CB1R and CB2R partial agonist with an agonist–antagonist activity [11]. In the last years, THC gained interest for its medical uses, including potential antiepileptic activity, analgesic properties, antiemetic effects in oncologic patients, and antispastic properties [12]. THC is characterized by high lipophilicity and rapidly distributes to tissues that are highly vascularized, including the brain. It is hydroxilated and transformed in the psychoactive metabolite 11-hydroxy-delta-9-THC (11-OH-THC), and through a successive oxidation in the nonactive compound 11-nor-9-carboxy-delta-9-THC (THCCOOH) [13]. THC produces its psychotomimetic effects primarily via stimulation of CB1R [14].

CBD is a nonpsychoactive chemical component of the plant cannabis but it is biologically active, even if it is not responsible for cannabis-induced euphoria or intoxication [15] (Figure 1). CBD influences cerebral activity and reduces seizures, modulates neurotransmission affecting behavior and social relations [16]. Although CBD has minimal binding-affinity for CB1R, it can influence the activity of CB1R in an indirect way [17]. Pre-clinical research showed CBD to be anticonvulsant in experimental animal models of epilepsy and to produce antipsychotic effects in experimental schizophrenia in humans [18,19]. Results from laboratory and human studies suggest that it could be a promising novel agent for central nervous system diseases, including schizophrenia [7] and epilepsy [20,21,22]. In humans, treatment with CBD is able to decrease the frequency of seizures showing a good risk/benefit profile [23,24]. CBD has a low affinity for cannabinoid receptors and only weakly antagonizes CB1 and CB2 receptors [25]; at CB1R level, it acts as a negative allosteric modulator [26]. CBD activity modulates several noncannabinoid targets, including the transient receptor potential subfamily V member 1 (TRPV1) cation channels [27], the G-protein-coupled receptor 55 [28], the enzyme fatty acid amide hydrolase [29], the peroxisome proliferator-activated receptor gamma (PPARγ) [30], serotonin 1A receptor (5HT1A) [31], and opioid receptors [32].

The therapeutic actions of THC and CBD are based on their antiemetic, analgesic, anti-inflammatory activities, and on their potential role in new treatments for neurodegenerative diseases [33,34]. Even though THC and CBD show differences in regard to their delivery in the brain, their lipophilicity and the variety of their properties lead us to consider them as possible new medicinal approaches to treat CNS diseases [35]. THC and CBD are almost exclusively the only cannabinoids investigated for their passage of the blood–brain barrier (BBB) and their delivery in the brain. Starting from the above exposed considerations, the aim of the present article is to show an overview about the cannabinoids THC and CBD crossing the BBB, and their successive brain disposition.

## 2. Methods: Search Strategy and Data Selection

Electronic databases, such as PubMed, Scopus, and ScienceDirect, were used for search by using “cannabinoids”, “cannabis“, “blood–brain barrier”, “brain delivery”, and “brain disposition” as the main keywords, starting from January 1971 to January 2020. In this review we collected and commented scientific articles published on peer-reviewed journals written in English language describing the relationship between pharmacokinetics of the cannabinoids THC and CBD, their crossing through the BBB and their brain delivery. Single topics were organized in the following sections: Introduction; Methods: search strategy and data selection; blood–brain barrier; cannabinoids and blood–brain barrier; cannabidiol and diseases involving blood–brain barrier breakdown; cannabinoid receptors and blood–brain barrier; cannabinoids pharmacokinetics and their delivery in the brain; cannabinoids and efflux pumps in the blood–brain barrier; conclusions.

## 3. Blood–Brain Barrier

The BBB is a functional interface between the anatomical structures forming the CNS and peripheral circulation including chemicals in blood [36]. Its primary function is the maintenance of CNS homeostasis and to protect the brain, preventing the entry from blood of pathogens or circulating chemicals, such as pathogens, which can act as neurotoxic or pro-inflammatory agents.

This is possible thanks to the characteristics of brain endothelium, which causes a critical limit thanks to the absence of fenestrations associated with the existence of tight junctions between the cells [37]. Beyond the absence of fenestrations brain vessels modulate exchange between circulation and CNS of molecules and cells. Other fundamental functions are the prevention of loss of essential substances through the control of transcellular movements of water and ions and the transfers of oxygen, CO2 and glucose necessary to cerebral cellular metabolism. For this reason, disorders involving BBB can cause ion dysregulation and alteration of homeostasis leading to brain dysfunction [38,39].

The architecture of the BBB is composed of endothelial cells lining brain microvessels, capillary basement membranes, and specific processes called end-feet extending from astrocytes to the basement membrane. Selective permeability of the BBB is guaranteed by brain microvascular endothelial cells connected together by intercellular tight junctions [40,41].

The BBB and microglia are the primary line of defense of the CNS. The BBB blood vessels are lined by endothelial cells. Endothelial cells are firmly linked together through tight junctions, surrounding basement membrane, and astrocyte end-feet together with pericytes embedded within the basement membrane support the structure of the BBB. The function of tight junctions is to reduce the paracellular pathway for the diffusion of hydrophilic solutes, with the result to allow the control of chemical substances present in the circulatory system, which can gain access to the brain [42].

## 4. Cannabinoids and Blood–Brain Barrier

The BBB works as an effective border between the CNS and systemic circulation [36,37] and microglia represents the main line of defense of the brain [43]. The BBB presence allows upholding an environment suitable for neuronal and glial cell function. Both neuronal cells and astrocytes are interconnected with microvessels forming the neurovascular unit regulating blood flow [44]. Other components of neurovascular unit are endothelial cells of BBB, myocytes, pericytes and elements of the extracellular matrix. Components of neurovascular unit are responsible for response to the request of neuronal supply triggering vascular changes such as vasoconstriction or vasodilation as necessary [45].

When neuroinflammation occurs, the BBB protective function can be reduced by an inflammatory reaction resulted from components of a neurovascular unit, especially from an immune-related cells involvement [46]. Moreover, breakdown of BBB facilitates brain infiltration of peripheral and central immune cells which leads to damage of neurons. Cannabinoids can influence positively brain immune response playing a role in prevention of BBB damage. In regard to this, it has been hypothesized that the activation of the endocannabinoid system could play a key role in preventing interactions between immune and endothelial cells and in neuroprotection through the keeping of tight junctions [47].

The BBB is prone to the consequences due to chronic systemic inflammation as it leads to brain inflammation and consequently can impair its integrity. In this context, interest has grown about the cannabinoids as potential anti-inflammatory drugs. In particular, it has been shown that CBD, which exhibits potent anti-inflammatory and immunosuppressive activity and modulates endothelial and epithelial barriers, could be useful to improve the deficit of the cognitive system and that this effect is due to protective effects towards the BBB [48].

Inflammation in the CNS implies the passage of leukocytes from blood through the BBB and the activation of local immunitary cells. By using the experimental model for multiple sclerosis of the Theiler’s murine encephalomyelitis virus-induced demyelinating disease, it has been proven that CBD can modify its detrimental consequences. In particular, it has been demonstrated that intraperitoneally (i.p.) CBD (5 mg/kg; once daily from days 1 to 7 post-infection), may exert protective effects by reducing the crossing of leukocytes from the systemic circulation by down-regulating the expression of the chemokines called C–C motif chemokine ligand 2 (CCL2) and C–C motif chemokine ligand 5 (CCL5), of interleukin-1 β, of vascular cell adhesion molecule-1 (VCAM-1), and by the attenuation of microglia activation [49]. These findings suggest that CBD could be part of a new strategic approach useful to treat inflammatory diseases of CNS.

Another experimental approach characterized by the utilization of a BBB model is represented by the use of human brain microvascular endothelial cells (BMEC) and human astrocyte co-cultures used to investigate the effects of CBD on BBB permeability, measured by transepithelial electrical resistance after ischemia induced by oxygen-glucose deprivation. In this model, CBD (10 μM) impeded the BBB permeability produced by four hours of oxygen-glucose deprivation. CBD protection was kept up to two hours after reperfusion and was suppressed by 100 nM of GW9662 (a PPARγ antagonist compound) and to a certain extent diminished by 300 nM of the compound WAY100135 (5-HT1A receptor antagonist). CBD protection was not changed by the addition of CB1R or CB2R antagonists or vanilloid or adenosine A2A receptors [50]. These data suggest that CBD, by triggering 5-HT1A and PPARγ receptors, could have a protective effect against BBB malfunction caused by ischemic events. Further, in vitro experiments on BMEC showed that CBD induces proliferation, migration, tubulogenesis, and trans-endothelial electrical resistance increase, indicating that CBD might be useful for the positive modulation of the BBB as a future treatment in ischemic stroke [51].

## 5. Cannabidiol and Diseases Involving Blood–Brain Barrier Breakdown

The treatment of CNS diseases is a major health challenge because of the current void of effective therapies, partially due to the obstacle to brain drug delivery represented by the BBB. From this point of view, since it is known that effective brain drug delivery should not be based only on passive targeting, active targeting through the use of nanomedicines into the brain has been investigated. In regard to this, the use of nanocarriers can be considered as an alternative to enhance the passage across the BBB [51]. Brain active targeting is formed on the transformation of nanoparticles with elements able to trigger transcytosis mediated by receptors in the brain by means of binding with molecules acting as transporters overexpressed by brain endothelial cells. In a recent study, lipid nanocapsules (LNCs), after incubation in a solution containing CBD (15 mg/mL) in a 3:1 (*v*/*v*) ratio, were investigated in vitro and in vivo (in mice) for their brain-targeting ability. Permeability experiments about biodistribution, performed with the cell-based in vitro BBB model of the human cerebral microvascular endothelial cell line D3, showed that the most brain-targeting capacity was realized by using the smallest LNCs decorated with cannabinoids. On the basis that a better effectiveness of transport in the brain is important for a therapy of brain diseases, the authors involved in the experiments suggested that small cannabinoid-decorated LNCs can represent a novel approach, inspiring the design and development of new therapies for CNS diseases [52].

The breakdown of the BBB architecture is a usual consequence of encephalitis induced by lipopolysaccharide (LPS) and may be produced by endothelial tight junction disruptions induced principally by cytokines. The augment of BBB permeability causes derivative lesions that exacerbate as long as the time period of a septic shock and aggravate the outcome [53]. Intravenous CBD (3 mg/kg) effects on inflammation and BBB damage in an in vivo model of sepsis induced by i.v. LPS (1 mg/kg) injection were investigated. Experiments demonstrated that the cannabinoid inhibited the arteriolar and venular vasodilation and leukocyte margination caused by LPS, abolished the enhancement of tumor necrosis factor-alpha and cyclooxygenase-2 expression, and expression of the enzyme inducible-nitric oxide synthase. Preservation of BBB integrity was also induced by CBD attenuation of LPS-induced modifications in the diameter and permeability of vessels and margination of leukocytes [54]. These results further suggest that CBD could be considered to develop a new strategic approach to protect BBB. Strategies for BBB protection are important because changes of this anatomical gateway have been found in people affected by CNS diseases, such as epilepsy, mental, and neurodegenerative disorders [55].

In patients affected by type 2 diabetes, the reduction of the integrity of the BBB is an early and serious condition, foregoing cognitive decline and in some cases dementia. It has been shown that CBD preserves damage of barrier endothelial function in a murine model of experimental diabetes induced by high glucose, thus suggesting that this cannabinoid could be able to reduce barrier alteration observed in this disease through the prevention of endothelial cell inflammation [56]. The same authors showed that CBD is able to prevent cellular margination and reduces the expression of adhesion molecules and chemotaxis by using laboratory models to investigate inflammation. For this reason, BBB could be thought as a CBD target to prevent potential dementia associated with diabetes and cannabinoids could be a new potential treatment option for this condition [57]. Chronicity of systemic inflammation contributes to cerebrovascular inflammation. As a consequence, dysregulation of regulatory proteinases occurs together with the disruption of the BBB’s tight junctions architecture. Furthermore, in subjects affected by type 2 diabetes, the risk of dementia is augmented and evidence suggests that chronic inflammatory processes and oxidative stress are involved in the disruption of the BBB, which precedes cognitive decline. In this context, recently, it has been proposed that positive response to cannabinoids on the cognitive system may be the result of preservative effects on the architecture and function of the BBB, thus suggesting that these compounds could produce a considerable advantage by protecting this structure. Moreover, despite of the small number of published studies investigating on the mechanisms playing a role in protective effects of CBD on BBB integrity, it has been suggested that this cannabinoid could stimulate the expression of proteins composing tight junctions [43].

## 6. Cannabinoid Receptors and Blood–Brain Barrier

CB1R is principally situated at the luminal side of the endothelium of BBB [58] and expression of brain CB1Rs was shown in astrocytes, microglial cells, pericytes [59]. Even though not at the same level of CB2R also is involved in neuroinflammation. It has been shown that neuronal expression of CB1R plays a key role to control experimental autoimmune encephalomyelitis [60] and mediates formation of interleukin-6 induced by the endocannabinoid anandamide in astrocytes in this experimental model [61].

Protective effects of cannabinoids in opposition to excitotoxicity are played principally at CB1R located on presynaptic axons, through inhibition of neurotransmitter release, included glutamate [62]. Other mechanisms suggested are opening of potassium channels followed by neuronal firing reduction, closing of voltage-sensitive calcium channels and reduction of cellular calcium influx [44]. It has been also shown that antagonism of CB1R reduces expression VCAM-1 induced by anandamide in cerebral endothelial cells and prevents adhesion and transmigration of leukocytes in the Theiler’s Murine Encephalomyelitis Virus experimental model [63].

CB2R is located at the abluminal side [58]. It has been demonstrated that CB2Rs are more expressed in human brain tissues of people affected by neurodegenerative pathologies, such as amyotrophic lateral sclerosis, multiple sclerosis, and Alzheimer’s disease [64,65]. The relationship between the BBB and potential changes of CB2Rs in brain endothelium has been studied in human immunodeficiency virus 1 (HIV-1) patients with neuroinflammation due to encephalitis. In these patients, the increase of expression of CB2Rs was detected in brain endothelial cells and microglial cells [66]. Successively, by using the experimental mouse model of LPS-induced encephalitis, other findings confirmed that CB2R agonists can reduce inflammatory responses at the BBB level. The highly selective CB2R agonists JWH133 and O-1966 administered i.p. at doses of 10 M and 5 M, respectively, reduced the adhesiveness of leukocytes to the surface of pial vessels and postcapillary venules of the deep cortical region, and they were efficaciously productive in the prevention of barrier breakdown induced by i.p. LPS at the dose of 6 mg/kg. In the same experiments, the addition of CB2R agonists enhanced trans-endothelial electrical resistance and the presence of tight junctions. Moreover, these compounds inhibited the surface expression of intercellular adhesion molecule-1 (ICAM-1) and VCAM-1 in human brain endothelial cells induced by pro-inflammatory mediators. These data taken together suggest that CB2R agonists could be used as a new therapeutic approach to protect the BBB against neuroinflammation [67].

## 7. Cannabinoids Pharmacokinetics and their Delivery in the Brain

The bioavailability of inhaled and oral THC is 20% and 6%, respectively. However, the quantity delivered in the brain is less than 1% of these percentages, indicating the high chemical potency related to the psychoactive effects of THC [68]. In early experiments, brain extraction of THC and 11-OH-THC was evaluated after intracarotid administration in rats of radiolabeled fractions using antipyrine as reference. The extraction proportion after five seconds was 66 ± 11% for THC and 70 ± 9% for 11-OH-THC, respectively, and 59 ± 4 for THC and 67 ± 8 for 11-OH-THC following 15 s. The larger 11-OH-THC presence in the brain could explain the pronounced central effects of this metabolite in comparison with the parent chemical despite being in the same concentration in plasma. Experiments also suggested that 11-OH-THC found in the liver after cannabis intake could have noteworthy brain effects [69].

After smoking, THC is absorbed rapidly with a bioavailability of 18–50% and reaches plasmatic peak in a few minutes. For this reason, many people consider smoking the preferred route for cannabis [70,71]. After inhalation through the smoke, THC is detectable in plasma after seconds with a peak in plasma after 3–10 min [72]. Inhalation with smoke of a cigarette with about 16–34 mg of THC produces peaks with mean concentrations ranging between 84.3 and 162.2 μg/L. Then, concentrations readily decrease down to 1–4 μg/L after three to four hours [73]. After oral administration, THC is absorbed more slowly and unpredictably with a peak in concentration generally obtained after one to three hours [70,74]. In this way, plasmatic THC has peaks of 0.58–12.48 μg/L, 2.7–6.3 μg/L, and 4.4–11 μg/L, after THC intake of 2.5, 15, and 20 mg [11]. In monkeys as in humans, the administration of THC in an intravenous way produced a peak in brain concentration following 15–60 min [75]. Following a single intramuscular dose of 30 mg in rats, brain availability of THC was reported to be 0.06% [76].

Data describing discrepancy between appearance of THC effects and its plasma concentration may be explained by pharmacokinetics of this compound. It has been suggested that rapid uptake associated with a slow release and storage of THC by neutral fat tissues in addition with blood–brain barrier limiting plasmatic concentration may represent a type of mechanism inherited phylogenetically with the aim to purchase protection for the brain against an exposure to fat soluble toxic agents [77].

THC is readily transformed to 11-OH-THC, which in turn is metabolized to the inactive metabolite THCCOOH. The plasma level readily falls while brain concentration rises. However, following the intravenous administration, at the peak time of psychoactivity, THC detected in the brain is approximately 1%. Despite the high perfusion of brain tissue, while THC is generally absorbed instantly in neutral fat, its brain delivery is slow and restricted. Low concentration found in the brain is thought to be caused by high perfusion velocity of THC quickly inside and outside of the brain. Furthermore, the brain entry of THC metabolite 11-OH-THC is more rapid and elevated in comparison to THC [11]. Thus, it can be deducted that 11-OH-THC significantly contributes to the psychoactive effects of THC, especially with oral intake. Furthermore, it has been also proposed that the pleasant sensory perception (“high”) of cannabis and the increase of the heart rate are interdependent with the concentration of THC and the number of functional THC receptor sites in the membrane lipid bilayer [78].

Evaluation of mean bioavailability of CBD in the plasmatic circulation after inhalation in cannabis users resulted in 31%, while the range was 11–45%. Following an oral dose of 40 mg, the plasma course of CBD over six hours was in the same range obtained with 20 mg of THC. However, five minutes after an intravenous administration in rats of doses of THC and CBD (1 mg/kg for each one), the concentration in the brain of unmodified CBD was found to be higher with respect to THC [11].

The THC and CBD delivery in the brain is not yet clearly described. One possible explanation is the variability of routes of administration used for cannabinoids, such as respiratory through smoking or vaporization, or oral through food or oil. The largest systemic THC bioavailability occurs when cannabis is smoked, with a serum peak reached within minutes [79]. Following oral administration of cannabis or cannabinoids, THC serum peaks are lower and occur after one to six hours [80]. 

Regarding nose-to brain route for administration of cannabinoids, CBD kinetics has been investigated. Absorption of CBD through the nose is rapid, within 30 s, with *T*_max_ ≤ 10 min. Cyclodestrins can be used to increase permeability of CBD in the brain because they interact with membranes of epithelium in the nose and are able to open temporarily the tight junctions [81].

Pharmacokinetics of i.p. CBD injection vs. oral administration has been studied. Injection to mice with a dose of 120 mg/kg of i.p. CBD produced higher concentrations both in plasma and brain than oral administration. Following i.p., in plasma and brain, maximum concentration (*T*_max_) of CBD was detected between one and two hours. Oral intake produced a faster peak in plasma (60 min) in comparison to brain (6 h). Drugs were no longer detected 24 h after administration. In the brain, the time that CBD took to reach the maximum concentration (*T*_max_) was low (*T*_max_ = 360 min) after oral administration, with an area under the curve (AUC) 0–6 h being 319 μg/g min compared to 1229 μg/g min following i.p. administration. Brain/plasma ratios evaluation based on AUC0–6 h was 0.84 and 0.51 after oral and i.p. administration, respectively, and thus indicating that i.p. and oral routes of administration produce a similar entry of cannabinoids in the brain. Pharmacokinetics profile investigated in rats gave similar results through oral and i.p. administration [35].

The brain cannabinoid concentration seems to be correlated with the amount of the cannabinoids in the inhaled marijuana. In a study performing the analysis of phytocannabinoids in mouse brain tissue through the use of liquid chromatography–mass spectrometry, mice were sacrificed 20 min following exposure to the smoke of 200 mg of Cannabis containing the following cannabinoids: CBD 0.93 mg, CBC 0.44 mg, and THC 8.81 mg. The analysis showed that average brain concentrations were CBD 21 ± 3.9 ng/g, CBC 3.9 ± 1.5 ng/g, THC 364 ± 74 ng/g, and 11-OH-THC 28 ± 5.9 ng/g [81]. These data show that there is a relationship between concentrations of cannabinoids CBD, CBC, and THC detected in the brain and the quantity of these chemicals found in the plant.

THC and CBD are highly lipophilic and quickly cross the BBB. Delivery of these cannabinoids into the brain following different routes of administration (subcutaneous, oral, and pulmonary) has been investigated in rats through gas chromatographic methods. To evaluate subcutaneous and oral ways, the cannabinoids THC and CBD, and the two chemicals together (THC/CBD) were administered one by one at the dose of 10 mg/kg singularly or in association. To investigate the pulmonary administration, the dose of 20 mg of THC or CBD or their association (20 mg for each substance in a 1:1 ratio) in ethanolic solution were dropped on the vaporizer. When THC and CBD were subcutaneously co-administered, THC brain concentration was found about four times higher and CBD concentration two times lower compared to the administration of each single cannabinoid. The cannabinoids inhalation brain peak level was detected at 15 min after administration and then gradually decreased. In this case, pharmacokinetics of THC/CBD combination was similar to those occurring when CBD or THC given singularly. The greatest THC brain concentration was found in a capacity of about one third in the serum. After oral administration, both THC and CBD showed a peak two hours after administration. When THC or CBD were given singularly, their level in the brain was high for two hours. Although the following oral administration serum peaks were similar to those obtained with inhalation, levels in the brain were three to six times higher, remaining at this level for four hours, thus indicating an accumulation of THC and CBD in brain tissue and explaining prolonged effects after oral cannabis intake. The product of THC metabolism, the psychoactive 11-OH-THC, was found following all ways of administration, showing the highest level with oral administration. Brain 11-OH-THC concentration reached about 200 ng/g, similar to brain THC concentrations following vaporization. Moreover, THC has been found in serum and the brain after administration of CBD singularly, which, if confirmed by other experiments, may open new views on pharmacokinetics of cannabinoids [82]. Overall, these findings show that cannabinoid serum and brain levels readily reach peak and decline following inhalation, while after subcutaneous and oral administration, long-lasting levels of cannabinoids occur, with oral intake producing the highest level in the brain.

Postmortem brain concentration of THC and of products of its metabolism (11-OH-THC, THCCOOH) were investigated in samples coming from eleven cases of lethal aviation accidents in which people have shown positivity for cannabinoids. The cannabinoid THC was found in six of ten brain samples at the concentration of 1.34–43.6 ng/g. In each THC positive brain sample, 11-OH-THC was also present at the concentration of 0.99–37.4 ng/g. In 9 of 10 brain samples, positivity for THCCOOH was detected (0.98–73.4 ng/g) [83].

## 8. Cannabinoids and Efflux Pumps in the Blood–Brain Barrier

Chemical substances that are not substrates of adenosine triphosphate-binding cassette (ABC) transporters will make more adequate drugs, because they do not show drug resistance, dependent at least on these proteins. Drug resistance is an important complication in the clinical management of neurological or psychiatric diseases, such as epilepsy and schizophrenia, with about 30% of not adequate responders in the treatment of patients [84]. Since several antipsychotic and anticonvulsants are considered substrates for P-glycoprotein (P-gp), it occurs that they are thrown out from the brain into the blood, which is an obstacle for brain accumulation [85]. ABC transporters have a key role in this kind of resistance [86]. They actively carry chemicals crossing biological membranes contributing to their disposal [87]. Among the ABC transporters, the most investigated are P-glycoproteins (P-gp or Abcb1) and breast cancer resistance proteins (Bcrp or Abcg2). These proteins are efflux pumps situated at biological barriers, including the BBB [88]. In epileptic and schizophrenic patients P-gp and Bcrp up-regulation at BBB level was found. This reduces the uptake of substances into the brain, determining the resistance to drugs [89,90].

**Figure 2:**
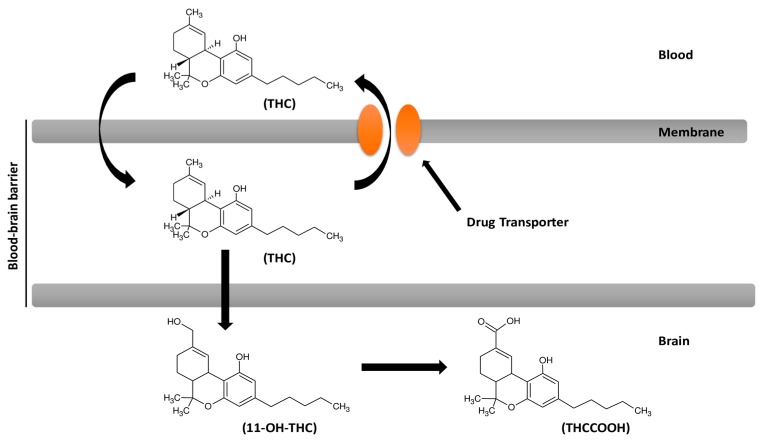
Influence of the drug transporter P-gp on passage through the blood–brain barrier and brain delivery of delta-9-tetrahydrocannabinol (THC). 11-OH-THC = 11-hydroxy-delta-9-THC (11-OH-THC); THCCOOH = 11-nor-9-carboxy-delta-9-THC (THCCOOH).

THC exhibits lower stimulation of adenosinetriphosphatase (ATPase) activity and it is effective in vitro in producing inhibitors of the Magnesium-ATPase (Mg-ATPase) and Sodium-ATPase (Na-ATPase). THC has affinity for P-gp and it is a P-gp substrate [91,92]. P-gp is expressed at endothelium of brain vessels and astrocytes, and it plays a key role in drug efflux at BBB level and influences individual responses of patients to CNS drugs [93]. High P-gp levels may reduce brain delivery of THC as well as decrease P-gp activity, which could cause its cerebral accumulation (Figure 2). In humans, polymorphism of phenotypes for the gene Multi-Drug Resistance 1 affects the development of dependence from cannabis. A more thorough investigation could be useful to increase knowledge on discrepancy observed in individual responses to THC effects [94].

It has been found that CBD, being a THC isomer, reduces P-gp and Bcrp transport, although this effect was found to be less congruous for the transporter P-gp [95]. In this regard, since inhibitors are often substrates, it is necessary to clarify if CBD is a substrate for one (P-gp) or both transporters (P-gp and Bcrp), and how this affinity influences brain crossing and delivery of this cannabinoid. In regard to this, experiments investigated CBD as a substrate for P-gp and Bcrp by using the experimental model of knockout mice for ABC transporter genes, comparing results of the cannabinoid with the drugs risperidone and 9-hydroxy risperidone, known to be substrates for P-gp [96]. Results of the study showed that CBD plasma concentrations were not higher in P-gp, Bcrp, or P-gp/Bcrp knockout mice compared with wild type mice. Therefore, since it has been suggested that CBD is not a substrate of P-gp or Bcrp and can represent a promising drug to be used for CNS diseases [94]. If we look at CBD as a therapeutic strategy for CNS diseases, and since P-gp or Bcrp do not obstacle the brain entry of CBD, as consequence, CBD may be free from the development of drug resistance when it is played by these ABC transporters.

## 9. Conclusions

Although previous publications on the passage of cannabinoids through the blood–brain barrier and their release in the brain were published, this is the first overview highlighting different themes connected to these topics. On the basis of multiple actions of phytocannabinoids THC and CBD and their lipophilicity, it has become evident that they may be taken into account for a deeper investigation on their potential role in new approaches for the treatment of CNS diseases. However, data from literature indicate that cannabinoids show dissimilarities in their pharmacodynamics and potency because of their different entry and disposition in the brain. While high P-gp levels may reduce brain delivery of THC, this review contains evidence demonstrating that CBD drug resistance is less likely to develop because it is not a substrate for the transporters P-gp or Bcrp, and, as a consequence, it represents a promising drug to use for CNS diseases.

## Abbreviations

ATPase	adenosinetriphosphatase
ABC	adenosine triphosphate-binding cassette
BBB	blood–brain barrier
BMEC	brain microvascular endothelial cells
Bcrp	breast cancer resistance protein
CBC	cannabichromene
CBD	cannabidiol
CB1R	cannabinoid receptor 1
CB2R	cannabinoid receptor 2
CCL2	C-C motif chemokine ligand 2
CCL5	C-C motif chemokine ligand 5
CNS	central nervous system
GPCR	G protein-coupled receptor
HIV	human immunodeficiency virus
ICAM-1	intercellular adhesion molecule-1
i.p.	intraperitoneally
LNCs	lipid nanocapsules
LPS	lipopolysaccharide
MDR1	Multi-Drug Resistance 1
Mg-ATPase	Magnesium-ATPase
P-gp	P-glycoprotein
PPARγ	peroxisome proliferator-activated receptor gamma
Na-ATPase	Sodium-ATPase
THC	delta-9-tetrahydrocannabinol
11-OH-THC	11-hydroxy-delta-9-THC
THCCOOH	11-nor-9-carboxy-delta-9-THC
TRPV1	transient receptor potential subfamily V member 1
5HT1A	serotonin 1A receptor
VCAM-1	vascular cell adhesion molecule-1
